# Silicate of Soda in Gonorrhœa

**Published:** 1874-09

**Authors:** 


SILICATE OF SODA IN GONORRHŒA.—At the Roosevelt Hospital, injections of the silicate of soda have been recently employed in the treatment of gonorrhœa, in both acute and chronic stages:

℞.—Silicate of soda, . . . . 20 grains.
Water, . . . . . . 8 ounces.—M.

The injections are given three times a day, with satisfactory results. No other treatment is combined with it.—*The Medical Record.*

